# Nuclear translocation and accumulation of glyceraldehyde-3-phosphate dehydrogenase involved in diclazuril-induced apoptosis in *Eimeria tenella* (*E. tenella*)

**DOI:** 10.1186/1297-9716-44-29

**Published:** 2013-05-07

**Authors:** Congcong Wang, Chunzhou Han, Tao Li, Dehao Yang, Xiaojiong Shen, Yinxin Fan, Yang Xu, Wenli Zheng, Chenzhong Fei, Lifang Zhang, Feiqun Xue

**Affiliations:** 1 Key Laboratory of Veterinary Drug Safety Evaluation and Residues Research, Chinese Academy of Agricultural Sciences, Shanghai Veterinary Research Institute, CAAS, Shanghai, 200241, PR China; 2 Hebei North University, Zhangjiakou, 075000, PR China

## Abstract

In mammalian cells, GAPDH (glyceraldehyde-3-phosphate dehydrogenase) has recently been shown to be implicated in numerous apoptotic paradigms, especially in neuronal apoptosis, and has been demonstrated to play a vital role in some neurodegenerative disorders. However, this phenomenon has not been reported in protists. In the present study, we report for the first time that such a mechanism is involved in diclazuril-induced apoptosis in *Eimeria tenella* (*E. tenella*). We found that upon treatment of parasites with diclazuril, the expression levels of GAPDH transcript and protein were significantly increased in second-generation merozoites. Then, we examined the subcellular localization of GAPDH by fluorescence microscopy and Western blot analysis. The results show that a considerable amount of GAPDH protein appeared in the nucleus within diclazuril-treated second-generation merozoites; in contrast, the control group had very low levels of GAPDH in the nucleus. The glycolytic activity of GAPDH was kinetically analyzed in different subcellular fractions. A substantial decrease (48.5%) in glycolytic activity of GAPDH in the nucleus was displayed. Moreover, the activities of caspases-3, -9, and −8 were measured in cell extracts using specific caspase substrates. The data show significant increases in caspase-3 and caspase-9 activities in the diclazuril-treated group.

## Introduction

*Eimeria tenella* (*E. tenella*), an obligate intracellular apicomplexan (coccidian) parasite, infects chickens and causes a severe form of coccidiosis. Coccidiosis is one of the most detrimental and lethal enteric diseases and is a major animal welfare and economic problem for the poultry industry. This rapidly developing intestinal disease presents with bloody diarrhoea and listlessness and can cause high mortality rates in affected flocks [1]. The *E. tenella* lifecycle is complex, involving both endogenous (schizogony and gametogony) and exogenous (sporogony) developmental stages [2]. During *E. tenella* 2nd generation, schizonts develop deep within the lamina propria and musculature, so their rupture and migration of merozoites back to the lumen eventually causes severe pathology.

Despite the advances in novel vaccines, coccidiosis is mainly controlled by chemotherapy to date. Diclazuril, one of the triazine-based agents effective against multiple types of coccidia in numerous hosts, has been used in poultry for a long time. In our previous study, we found that after treatment with diclazuril, merozoites showed classic apoptotic features in the control/treatment group [3]. Apoptosis is a tidy, regulated process by which the apoptotic cell quietly commits suicide without disturbing its neighbors, and the corpse remains neatly packaged [4]. So far, apoptosis has been described in all invertebrate and vertebrate multicellular organisms studied [5]. In multicellular organisms, one primary mechanism for this cell dismantling process involves the activation of caspases through mitochondrial and death receptor-mediated mechanisms that is caused by a diverse range of stimuli including extracellular or intracellular inducers [6].

Recently, another mechanism of apoptotic cell death has been reported. Glyceraldehyde-3-phosphate dehydrogenase (GAPDH; EC 1.2.1.12), a key enzyme in glycolysis and gluconeogenesis, has long been regarded merely as a highly conserved protein that regulates the conversion of glyceraldehyde-3-phosphate to 1, 3-diphosphoglycerate, which has a central role in energy production. Recent studies suggest that GAPDH is a multifunctional protein that plays a role in membrane transport, microtubule assembly, and so on [7,8]. In addition to these functions, one of the most intriguing roles of GAPDH is its integral role in the death of one or more cell lines [9,10]. To date, there have been numerous reports of apoptosis in unicellular intestinal parasites that have morphological and biochemical similarities to cell death in multicellular organisms [11-14]. However, this mechanism has not been reported in protists. Here, we report the first evidence of nuclear translocation and accumulation of glyceraldehyde-3-phosphate dehydrogenase during diclazuril-induced apoptosis in *E. tenella*.

## Materials and methods

### Reagents

Diclazuril (> 99%, No. 20110118) was provided by Shanghai Veterinary Research Institute, Chinese Academy of Agriculture Sciences (CAAS).

### Second-generation merozoite collection

A slightly modified version of a previously described procedure was used [3,15]. After receiving permission from a local committee of the Faculty of Veterinary Medicine and conforming to the guidelines of Institutional Animal Care and Use Committee of China, the experiment was carried out as follows. A total of 400 one-day-old Chinese yellow broiler male chickens were purchased from the Hatchery of Huizhong, Shanghai, China. The chickens were reared under standard sanitary conditions and given ad libitum access to water and an artificially prepared diet containing no antihelmintics or anticoccidial drugs. To maintain a temperature of 30°C, an electric radiator and ventilation fans were used. At 14 days of age, chickens were randomly assigned to one of two groups of 200 chickens each, and each group was further subdivided into four blocks of 50 chickens each as biological replicates. Each chicken was inoculated with 8 × 10^4^ oocysts suspended in 1 mL of water by oral gavage, and two treatment conditions were employed: (1) chickens were challenged with *E. tenella* oocysts and provided with normal feed as the control group or (2) chickens were challenged with *E. tenella* oocysts and provided with 1 mg/kg diclazuril in feed from 96 h to 120 h after inoculation as the treatment group. All of the chickens were given unlimited access to a standard diet and water. The chickens were euthanized at time points up to 120 h after infection, and the second-generation merozoites were prepared according to the method described by Liu et al. [16]. Briefly, the merozoites were prepared by enzymatic digestion, centrifugation, erythrocyte disruption, and Percoll density gradient centrifugation.

### Real-time PCR

Total RNA was extracted from the second-generation merozoites with TRIzol reagent (Invitrogen, USA) following the manufacturer’s instructions. To avoid DNA contamination, the extracted RNA was treated with RNase-free DNase I (40 U/mg RNA, Takara, China) and purified using the RNeasy Mini Kit (Qiagen, Germany) according to the manufacturer’s instructions. cDNA was synthesized from purified RNA using the SuperScript™ II Reverse Transcriptase kit (Invitrogen, USA) and the random hexamer primer pd (N) _6_ PCR amplification of the *gapdh* gene (GenBank: HQ317455) was performed using the primers (Sense 5′- CGCCACCTAAGGACGATA -3′; Antisense 5′- TGCCAAGGGAGCCAAGCA-3′) with SYBR® Premix EX Taq™ (Perfect Real Time) kit (Takara, China) on the RG-3000A real-time PCR system (RoterGene, USA). The housekeeping gene β-actin was amplified using the primers (Sense 5′-GGATTGCTATGTCGGCGATGA-3′; Antisense 5′-ACACGCAACTCGTTGTAGAAAGTG-3′). The RT-PCR protocol included an initial denaturation at 95°C for 10 s, followed by 40 cycles consisting of denaturation at 95°C for 5 s, annealing at 60°C for 10 s, and extension at 72°C for 15 s at the end of the PCR. The specificity of amplification was confirmed using a melting curve and electrophoresis analysis. The Real-time PCR data were analyzed with a normalized gene expression method (2-ΔΔCT) [17]. Each reaction was performed in triplicate, and the entire experiment was carried out in triplicate.

### Western blot

For the preparation of polyclonal antibody against GAPDH, 5-week-old female BALB/c mice were immunized by i.p. injection with 100 μg per mouse of the recombinant *E. tenella* GAPDH protein (prepared by our laboratory) emulsified in FCA (Sigma USA). The mice were boosted 2 weeks later and re-boosted three times at 1-week intervals with 100 μg purified recombinant protein emulsified in Freund incomplete adjuvant (Sigma, USA). Sera were obtained 1 week after the final immunization. The antibody titer was determined by ELISA.

The second-generation merozoites were suspended in PBS and lysed by ultrasonic disruption. The protein concentration was determined using a Quick Start ™ Bradford Protein Assay Kit 2 (BioRad, China) Lysate containing equal amounts (40 μg) of protein that were loaded onto each lane of an SDS-polyacrylamide gel for electrophoresis and subsequently transferred onto polyvinylidene difluoride membranes. The membranes were blocked in 10% skim milk, then incubated with GAPDH antibody (1:8000 dilution) or tubulin antibody (1:1000 dilution, Beyotime, China) for 2 h at 37°C, and finally incubated with horseradish peroxidase-conjugated Goat anti-mouse IgG (1:5000 dilution, Jacksonimmuno, USA) for 1 h at 37°C. Peroxidase activity was assayed using the BeyoECL plus Kit (Beyotime, China). For comparative quantitative protein expression profile analysis, the membranes were scanned, and the resulting images were analyzed using the ImageJ 1.46 software (National institutes of Health, USA).

### Subcellular fractionation

Nuclear and cytoplasmic fractions were prepared using NE-PER Nuclear and Cytoplasmic Extraction Reagents (Pierce) according to the manufacturer’s protocol.

### Immunofluorescence assay

To carry out the Immunofluorescence assay, freshly-obtained second-generation merozoites were fixed on glass slides using 2% paraformaldehyde for 30 min at room temperature (RT), washed three times in PBS, permeabilized with 1% Triton X-100 in PBS for 15 min at RT, washed again, and blocked with 2% BSA in PBS overnight at 4°C. After three washes, the slides were incubated successively with 1:1000 diluted antibodies against GAPDH and 1:400 diluted FITC (fluorescent isothiocyanate) conjugated goat anti-mouse IgG (Beyotime, China) for 1 h at 37°C in the dark. The slides were incubated with DAPI (1 μg/mL, Beyotime, China) for 30 min, and finally, 50 μL of Antifade Mounting Medium (Beyotime, China) was added for examination under fluorescence microscopy [15].

### GAPDH enzyme activity

The enzyme activity was measured according to the method described by Kanwar and Kowluru [18]. The enzyme activity was measured spectrophotometrically in a final assay volume of 100 μL containing 50 mmol/L triethanolamine buffer (pH 7.6), 50 mmol/L arsenate, 2.4 mmol/L glutathione, 250 μmol/L NAD, and cytosolic/nuclear protein (5 μg protein). The assay mixture was preincubated for 5 min at 37°C, and the reaction was initiated using 100 μg/mL glyceraldehyde-3-phosphate. Increases in NADH production were monitored at 340 nm. GAPDH activity was expressed as the difference in absorbance in the presence/absence of glyceraldehyde-3-phosphate.

### Caspase activity assay

The activities of caspase proteins were measured using the Caspase-Glo® 9 Assay (Promega), the Caspase-Glo®3/7 Assay (Promega), and the Caspase-Glo® 8 Assay (Promega) according to the manufacturer’s protocol.

### Statistical analysis

Statistical analysis was performed using the Student’s *t*-test. Values of *P* < 0.05 or *P* < 0.01 were considered significant.

## Results

### The expression of GAPDH is increased in apoptotic merozoites

**Figure 1:**
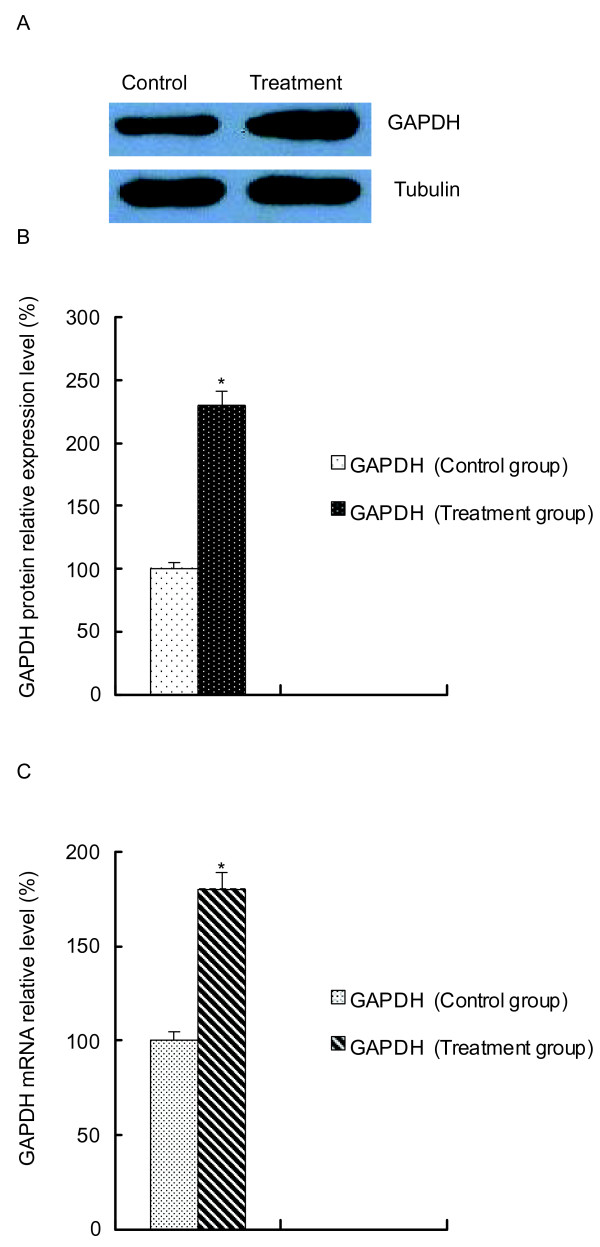
**The overexpression of GAPDH in apoptotic second-generation merozoites induced by diclazuril. A**. Representative Western blots showing the protein level of GAPDH. Tubulin serves as a loading control. **B**. The relative quantification of GAPDH protein level. The results were expressed as the mean ± SD of 3 experiments. *P < 0.05 vs. control group. **C**. Quantitative real-time PCR results showing the relative mRNA level of GAPDH. The results were expressed as the mean ± SD of 3 experiments performed in triplicate.*P < 0.05 vs. control group.

Previously, we showed that diclazuril induces merozoite apoptosis in *E. tenella*[3]. However, the exact mechanism by which this drug induces apoptosis is not yet completely clear. Using two-dimensional gel electrophoresis, we found that the expression level of GAPDH was significantly affected by diclazuril treatment (unpublished data). In the present study, Western blot analysis was used to determine changes in GAPDH expression. A significant increase (about 130%) in the expression of GAPDH protein was observed in apoptotic merozoites, as shown in Figure 1a and b. Furthermore, the quantity of *gapdh* mRNA was measured using Real time PCR. These apoptotic cells also expressed higher levels of *gapdh* mRNA than the non-diclazuril-treated group (Figure 1c), implying de novo synthesis of GAPDH.

### Nuclear translocation and accumulation of endogenous GAPDH after induction of apoptosis by diclazuril

**Figure 2:**
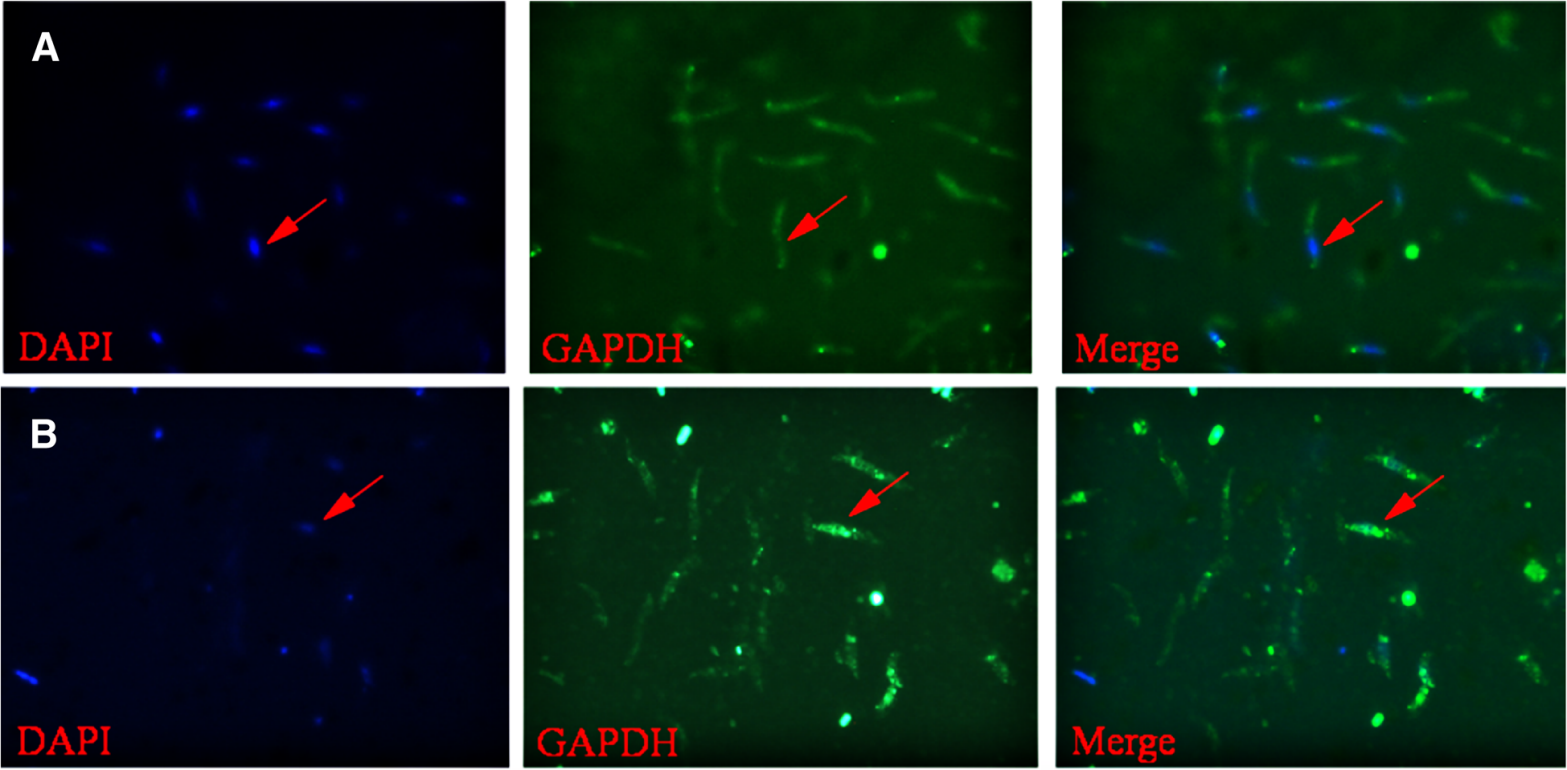
**Nuclear translocation and accumulation of endogenous GAPDH after diclazuril treatment.** A difference in subcellular localization of endogenous GAPDH was observed in second-generation merozoites between the control group and the Treatment group. The distribution of endogenous GAPDH was detected by fluorescence microscopy; the GAPDH immunostaining was almost undetectable in the nuclei of second-generation merozoites of the control group (**A**). In contrast, the considerable GAPDH immunostaining (green) appeared to be in the nuclei of second-generation merozoites of the treatment group (**B**). DAPI staining (blue) to detect parasite nuclei. Merge is GAPDH immunostaining/DAPI staining overlay. Arrows show nuclei localization.

We examined the distribution of endogenous GAPDH by fluorescence microscopy. As shown in Figure 2, the GAPDH immunostaining was almost undetectable in the nuclei of control second-generation merozoites (Figure 2a). In contrast, considerable GAPDH immunostaining appeared in the nuclei of diclazuril-treated second-generation merozoites (Figure 2b).

### The glycolytic activity of GAPDH in the nuclear and cytoplasmic subfractions

**Figure 3:**
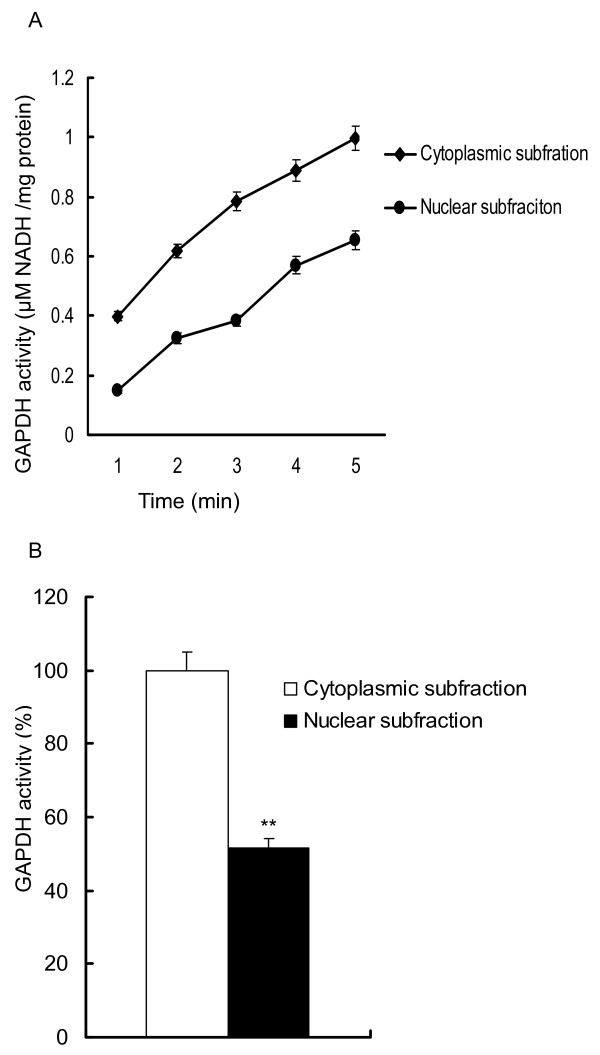
**The glycolytic activity of GAPDH in nuclear and cytoplasmic subfractions. A**. The glycolytic activity of GAPDH was quantified by measuring the production of NADH at 340 nm for 5 min (μM NADH/mg protein). **B**. The relative GAPDH glycolytic activity. The results are expressed as the mean ± SD of 3 experiments performed in triplicate. **P < 0.01 vs. control group.

The involvement of GAPDH has been implicated in multiple types of apoptotic paradigms [19-23]. One primary mechanism through which GAPDH executes its role during apoptosis is translocation to the nucleus from the cytoplasm [24]. The translocation of GAPDH to the nucleus and its accumulation there were accompanied by the formation of subcellular GAPDH: neuronal protein complexes resulting in the formation of non-glycolytic oligomers from enzymatically active GAPDH tetramers [10,25]. To measure changes in glycolytic activity, the enzyme activity was studied by the kinetics method in different subcellular fractions prepared from the merozoites of the treatment group. The results were consistent with previous reports in that the GAPDH catalysis displayed a significant decrease in activity of 48.5% in nuclear fractions compared to cytoplasmic fractions (Figure 3).

### Higher increased caspase-3 and caspase-9 activity in apoptotic merozoites

**Figure 4:**
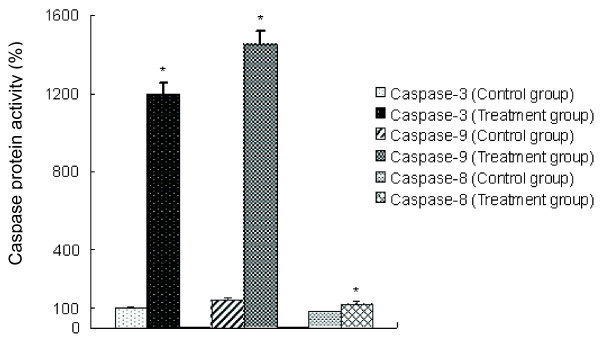
**Larger increases in caspase-3 and caspase-9 activity in apoptotic merozoites in the treatment group.** The activities of caspase proteins in merozoites in the control group and the treatment group were measured using specific caspase kits. *P < 0.05 vs. control group.

Some published reports have suggested that the nuclear translocation of GAPDH is related to the activation of caspase-3 protein during apoptosis in mammalian cells and that it might be an early event before the apoptotic cascade is inevitable [26,27]. Previous studies showed caspase-like activity during apoptosis in protozoons [28,29]. However, the relationship between GAPDH and caspase activity has not been reported in protozoons, and no reports have examined the apoptotic pathways involved in protozoon apoptosis. Thus, the activities of different caspase proteins were measured using specific caspases substrates. The data show that treatment of merozoites with diclazuril resulted in a conspicuous increase in the activities of caspase-3 and caspase-9, but not caspase-8 (Figure 4).

## Discussion

Generally, GAPDH is considered as a housekeeping enzyme and is often used as an invariant control for gene expression studies [30,31]. In the last decade, some reports have shown that in addition to the well-described function in glycolysis, GAPDH is a complex multifunctional protein involved in a number of cellular processes, such as cell proliferation and apoptosis in mammalian cells [32-36]. We assessed whether this protein was involved in such cellular processes using the model of apoptosis induced by diclazuril in *E. tenella*. To our knowledge, the present study is the first report demonstrating the overexpression and subsequent nuclear accumulation of GAPDH in apoptotic protozoon.

Lal et al. investigated the proteome of four *E. tenella* life cycle stages [37]. They found that GAPDH plays an important role in basic cell catabolic processes as a housekeeping glycolytic enzyme in four life cycle stages of *E. tenella* (unsporulated oocyst, sporulated oocyst, sporozoite and second generation merozoites). In second-generation merozoites, it has been demonstrated that the parasite uses anaerobic glycolysis during schizogony but transitions to aerobic metabolism before merozoite release. Nevertheless, we examined GAPDH mRNA and protein levels in second-generation merozoites after diclazuril treatment. The result of RT-PCR analysis revealed considerable increases in *gapdh* mRNA, and Western blot analysis demonstrated that GAPDH protein levels paralleled GAPDH mRNA expression. These results were consistent with previous reports of substantial up-regulation of GAPDH during apoptosis. Ishitani et al. first described that in cultured cerebellar granule cells and cortical neurons undergoing spontaneous apoptosis, the expression of GAPDH was increased prior to apoptosis [38]. Further investigation using the *gapdh* knockdown strategy in various cell types confirmed the generality of the role of GAPDH in some mammalian cells [8].

Usually, the major action of GAPDH takes place in the cytoplasm during glycolysis [39]. In the present study, the results of our fluorescence microscopy show that immunoreactive GAPDH proteins largely appeared to be abundantly distributed in the nucleus after diclazuril treatment. Although the role of GAPDH in the nucleus during apoptosis has not been elucidated, it seems that the nuclear translocation of this protein is a requirement for apoptosis to proceed [40]. On the contrary, lacking a common nuclear localization signal (NLS), GAPDH cannot enter the nucleus by itself because of its size. In mammalian cells, it is well documented that GAPDH binds the NLS-bearing siah-1 (an E3 ubiquitin ligase), forming a complex that subsequently promotes translocation of GAPDH from the cytosol to the nucleus. It is postulated that the NLS on siah-1 facilitates the movement of this complex into the nucleus [41]. A previous study using expressed sequence tags (EST) derived from a merozoite cDNA library in *E. tenella* suggested that a ubiquitin ligase ortholog exists in the *E. tenella* genome [42]. Unfortunately, we failed to identify this complex using co-immunoprecipitation. The mechanism by which GAPDH translocates to the nucleus in *E. tenella* needs further investigation.

Previous studies indicate that changes in subcellular localization may be accompanied by various post-translational modifications (phosphorylation or ADP ribosylation), which lead to changes in GAPDH activity [39]. The glycolytic activity of GAPDH in the nuclear subfraction was much lower compared to the cytoplasmic subfraction in cells treated with diclazuril, despite a significant increase in expression in the nuclear subfraction thus agreeing with the latter data.

In metazoan cells, one primary mechanism for the execution of apoptosis is associated with the action of caspases [43]. The activity of caspases is crucial for the initiation of apoptotic DNA fragmentation, chromosome condensation and other apoptotic cellular phenotypes [44,45]. In our previous work, we demonstrated that the diclazuril treatment in second-generation merozoites of *E. tenella* results in classic morphological and biochemical similarities to apoptosis in higher vertebrate multicellular organisms [3]. However, there are no reports on the role of caspases in *E. tenella* so far. Thus, in this study, we tested caspase-like activity using specific substrates for caspase-3, -9 and −8. The activities of caspase-3,-9, and −8 were obviously detected in both experimental groups (control/treatment group), suggesting that caspases-3,-9, and −8 were involved in apoptosis in *E. tenella*, but treatment with diclazuril led to much larger increases in the activities of caspase-3 and caspase −9, indicating that diclazuril activates the intrinsic caspase-9 apoptotic pathway, not the extrinsic caspase-8 apoptotic pathway. Usually, the activity of caspase-9 is connected with the mitochondrial pathway in apoptosis [29]. This finding was in line with our previous results showing the onset of diclazuril induced apoptosis in *E. tenella*, as evidenced by the lethal effects of diclazuril on the mitochondrial transmembrane potential in merozoites [3].

In summary, our current study conclusively demonstrates that the nuclear translocation and accumulation of glyceraldehyde-3-phosphate dehydrogenase is involved in diclazuril-induced apoptosis in *E. tenella*. This process is accompanied by the activation of the intrinsic caspase-9 pathway, not the extrinsic caspase-8 pathway. Although the precise mechanism responsible for the involvement of GAPDH in apoptosis is unclear, these findings shed new light on the pathogenesis of *E. tenella* during its interaction with diclazuril. Further investigation into this aspect might contribute to new drug targets for chemotherapeutic intervention in this parasite.

## Competing interests

The authors declare that they have no competing interests.

## Authors’ contributions

CCW carried out most of the experiments; TL designed the methods and experiments, interpreted the results and finished the discussion. DHY, XJS, YXF, YX, WLZ, CZF and LFZ prepared the merozoites and other materials, and partly worked on the experiments. FQX was responsible for overall supervision, and participated in coordination. All authors read and approved the final manuscript.
